# Case Report: Identification of a CARD8 variant in all three patients with PFAPA syndrome complicated with Kawasaki disease

**DOI:** 10.3389/fped.2024.1340263

**Published:** 2024-03-05

**Authors:** Haruhiko Nakamura, Atsuo Kikuchi, Hideyuki Sakai, Miki Kamimura, Yohei Watanabe, Ryoichi Onuma, Jun Takayama, Gen Tamiya, Yoichi Mashimo, Ryota Ebata, Hiromichi Hamada, Tomohiro Suenaga, Yoshihiro Onouchi, Satoru Kumaki

**Affiliations:** ^1^ Department of Pediatrics, National Hospital Organization Sendai Medical Center, Sendai, Japan; ^2^ Department of Pediatrics, Tohoku University School of Medicine, Sendai, Japan; ^3^ Department of Rare Disease Genomics, Tohoku University School of Medicine, Sendai, Japan; ^4^ Department of AI and Innovative Medicine, Tohoku University School of Medicine, Sendai, Japan; ^5^ Tohoku Medical Megabank Organization, Tohoku University, Sendai, Japan; ^6^ Statistical Genetics Team, RIKEN Center for Advanced Intelligence Project, Tokyo, Japan; ^7^ Department of Public Health, Chiba University Graduate School of Medicine, Chiba, Japan; ^8^ Department of Pediatrics, Chiba University Graduate School of Medicine, Chiba, Japan; ^9^ Department of Pediatrics, Tokyo Women’s Medical University Yachiyo Medical Center, Yachiyo, Japan; ^10^ Department of Pediatrics, Wakayama Medical University, Wakayama, Japan

**Keywords:** CARD8, inflammasome, NLRP3, inflammatory dysregulation, Kawasaki disease, PFAPA syndrome

## Abstract

**Background:**

Periodic fever, aphthous stomatitis, pharyngitis, and cervical adenitis (PFAPA syndrome), and Kawasaki disease (KD) are both considered to be disorders of the innate immune system, and the potential role of inflammasome activation in the immunopathogenesis of both diseases has been previously described.

**Case presentation:**

Herein, we report the clinical courses of three patients who presented a rare combination of PFAPA syndrome and KD. Two patients who presented KD later developed the PFAPA syndrome, of whom one developed recurrent KD 2 years after the initial diagnosis. The third patient developed KD one year after the onset of PFAPA syndrome. The presence of both of these conditions within individual patients, combined with the knowledge that inflammasome activation is involved in both PFAPA syndrome and KD, suggests a shared background of inflammatory dysregulation. To elucidate the mechanism underlying shared inflammatory dysregulation, we investigated the roles of Nod-like receptors (NLRs) and their downstream inflammasome-related genes. All the patients had a frameshift variant in *CARD8* (CARD8-FS). A previous study demonstrated a higher frequency of CARD8-FS, whose product loses CARD8 activity and activates the NLRP3 inflammasome, in patients with the PFAPA syndrome. Additionally, the NLRP3 inflammasome is known to be activated in patients with KD. Together, these results suggest that the CARD8-FS variant may also be essential in KD pathogenesis. As such, we analyzed the *CARD8* variants among patients with KD. However, we found no difference in the variant frequency between patients with KD and the general Japanese population.

**Conclusions:**

We report the clinical courses of three patients with a rare combination of PFAPA syndrome and KD. All the patients had the CARD8-FS variant. However, we could not find a difference in the variant frequency between patients with KD and the general Japanese population. As the frequency of KD is much higher than that of PFAPA among Japanese patients, and the cause of KD is multifactorial, it is possible that only a small portion of patients with KD harbor CARD8-FS as a causative gene.

## Introduction

Periodic fever, aphthous stomatitis, pharyngitis, and cervical adenitis (PFAPA) syndrome is the most common recurrent self-limiting fever affecting young children, with sufferers being asymptomatic in the intervals between flares (1). Marshall first reported PFAPA in 1987 (2), and thereafter, the Thomas criteria for diagnosis have been created and widely disseminated (3). Most patients’ symptoms resolve spontaneously before adulthood (1). PFAPA syndrome is considered an autoinflammatory disorder of unknown etiology, but a genetic basis has been assumed because of the observance of familial links and similarities to other autoinflammatory diseases (4). Kawasaki disease (KD), first reported by Tomisaku Kawasaki in 1967 (5), is an acute self-limiting systemic vasculitis of unknown etiology that presents in early childhood (5, 6). The main symptoms are fever for more than five days, rash, edema of the hands and feet, lip erythema, strawberry tongue, bilateral conjunctivitis, and cervical lymphadenopathy; the criteria for diagnosis involve the fulfillment of these symptoms (7). Genetic factors are presumed to be important in KD due to observed racial differences and family histories, such as a high incidence among siblings (6). The immune dysfunction linked with KD is thought to involve an abnormal innate immune response (6). A previous report showed a higher prevalence of KD among patients with PFAPA syndrome (8). The existence of these two conditions within a single patient suggests a possible genetic predisposition for inflammatory dysregulation. As the potential role of inflammasome activation in the immunopathogenesis of both PFAPA syndrome and KD has been previously described in the literature (9–11), in the present study, we focused on the role of Nod-like receptors (NLRs) and inflammasome-related genes in the pathogenesis of PFAPA syndrome and KD.

## Case presentation

All patients discussed in this study were recruited from the Department of Pediatrics of Sendai Medical Center between January 1, 2016, and December 31, 2019. PFAPA syndrome and KD were diagnosed based on previously established diagnostic criteria (3, 7). KD was detected in three cases (8.3%) among 36 patients with PFAPA syndrome treated in this department.

### Case 1

A 3-year-old boy was admitted to our hospital with a 5-day history of fever (38 °C), rash, injected conjunctiva, and red lips. The patient's medical history was unremarkable, although his maternal uncle had a history of recurring tonsillitis. No other family history of autoinflammatory disorders, autoimmune diseases, or KD was noted. Laboratory findings indicated enhanced inflammatory response. The patient was diagnosed with KD on the sixth day of fever, treated with one dose of intravenous immunoglobulin (IVIg; 2 g/kg), and initiation of oral aspirin therapy. Echocardiography revealed no cardiovascular abnormalities. One month after recovering from KD, recurrent episodes of fever associated with pharyngitis were noted. Subsequently, febrile episodes lasting 4–5 days at 39–40 °C occurred every month. Between febrile episodes, the patient was healthy and his laboratory data, including inflammatory responses, returned to normal. He was subsequently diagnosed with PFAPA syndrome, and cimetidine (20 mg/kg/day) was initiated. However, cimetidine treatment was discontinued due to the development of a skin rash that resembled erythema multiforme. He was then treated with oral prednisolone (1 mg/kg) at the onset of the fever, which resolved the fever within a few hours. When the patient was 5 years old, he took prednisolone for three consecutive days, but it was ineffective. Subsequently, his eyes became reddish, and he developed a rash on the limbs, edema of the hands and feet, and cervical lymphadenopathy. He again fulfilled the diagnostic criteria (five out of six) for KD, and was treated with IVIg and aspirin. His recurrent episodes of fever persisted even after the recurrence of KD. A series of echocardiograms revealed no cardiovascular abnormalities.

### Case 2

A 1-year and 5-month-old girl was diagnosed with KD on the fourth day after presentation with persistent fever, rash, swollen cervical lymph nodes, red lips, and edema of the hands and feet. Her maternal uncle was diagnosed with PFAPA syndrome. The patient fulfilled all six criteria for KD and was treated with IVIg (2 g/kg) and oral aspirin on the fourth day. However, her fever persisted, requiring additional IVIg on the eighth day. Echocardiography revealed no cardiovascular abnormalities. Subsequently, she experienced recurrent fever each month, and was diagnosed with PFAPA syndrome. She was treated with cimetidine but showed no improvement. The fever subsided within a few hours following the administration of oral prednisolone.

### Case 3

A 3-year-old boy presented with monthly fever and was diagnosed with the PFAPA syndrome. He was born prematurely at 32 weeks of gestation, with a birth weight of 1,739 g, and had periventricular leukomalacia. The patient's family history was unremarkable. At the age of four, the patient experienced persistent fever, conjunctival hyperemia, and dilatation of the left main trunk coronary artery (Z-score = 3.31). Subsequently, the patient was diagnosed with incomplete KD. We opted for oral aspirin over IVIG for treatment based on the patient's pre-existing defervescence trend at diagnosis. His fever was relieved with the therapy, and his coronary arteries spontaneously normalized. After recovering from KD, the patient began experiencing monthly fevers again. Therefore, cimetidine was initiated, and the interval between the fever episodes was gradually extended.

#### Identification of CARD8-Fs in all three patients

We performed whole-exome sequencing of the three patients and their parents. A heterozygous TT insertion in exon 8 of *CARD8* (NM_001184900) was identified in all three patients and their mothers. This 2-bp insertion was identical to CARD8-FS, annotated as rs140826611 in the Single Nucleotide Polymorphism database (db SNP), a frameshift variant previously shown to be associated with PFAPA syndrome (Table 1) (12, 13). In addition, we identified a heterogenous missense mutation p.R32C at position rs76935241 in *NLRP11* in two patients and their parents, which was absent in the third patients. We further identified the heterogeneous missense mutations, p.V1059M and p.L155H (rs2301582 and rs12150220, respectively) in *NLRP1* and p.T529A (rs34804158) in *NLRP2*, but each was present in only one patient (Table 1). In summary, none of the variants within the genes described in the Table 1, other than *CARD8,* were shared among the three patients. These results suggest that the CARD8-FS variant plays an essential role in the pathogenesis of KD; thus, we analyzed CARD8-FS in 752 patients with KD. We found that the frequency of CARD8-FS among patients with KD was 13.0%, equivalent to the general Japanese population (12.9%) (14).

**Table 1:** Variants in *NLR* and inflammasome-related genes.

Gene	rs number	AA change	MAF^a^	Case 1			Case 2		Case 3		
				Father	Mother	Patient	Mother	Patient	Father	Mother	Patient
*NLRP1*	rs2301582	p.V1059M	0.0248	Hetero		Hetero					
*NLRP2*	rs34804158	p.T529A	0.0656	Hetero		Hetero					
*NLRP2*	rs12973968	(c.2538–4G > C)	0.0481	Hetero		Hetero					
*NLRP11*	rs76935241	p.R32C	0.0600		Hetero		Hetero	Hetero	Hetero		Hetero
*NLRC5*	rs371576531	p.V800I	0.0003		Hetero	Hetero					
*NWD1*	rs11671361	p.D1541V	0.1084						Hetero		Hetero
*NLRP1*	rs12150220	p.L155H	0.0375	Hetero		Hetero					
*NAIP*	rs200427852	p.E158Q	0.0114				Hetero	Hetero			
*CARD8*	rs140826611	p.V148fs	0.1299		Hetero	Hetero	Hetero	Hetero		Hetero	Hetero

AA, amino acid; MAF, minor allele frequency; Hetero, heterozygote; *NLR*, nod-like receptor.

^a^ ToMMo4.7KJPN.

## Discussion

Herein, we report three patients who met the clinical criteria for both PFAPA syndrome and KD (2, 4). Two of these patients who presented with KD later developed PFAPA syndrome, with one developing recurrent KD 2 years after its initial diagnosis. The third patient developed KD one year after the onset of PFAPA syndrome. The co-occurrence of these two conditions within a single patient suggest a common genetic link for inflammatory dysregulation. Similar cases have been reported previously (8, 15). However, the common background of FPAPA syndrome and KD has not been analyzed.

PFAPA syndrome and KD share many common symptoms, including fever, cervical lymphadenopathy, and oral cavity mucosa redness (1, 7). The phenotypic similarities between PFAPA syndrome and KD may be partially explained by the common immunobiological processes associated with inflammasome activation. Elevated levels of sIL-1β and IL-18, the cytokine signatures associated with inflammasome activation, are found in both patients with PFAPA syndrome and KD (10, 16, 17). Additionally, KD was reported to occur in 4.7% of patients with PFAPA syndrome, which was much higher than the incidence of KD (0.02%) among children younger than 5 years of age in San Diego County, USA (8). In our study, KD occurred in three cases (8.3%) among 36 patients with PFAPA syndrome, which was significantly higher than the incidence of KD (0.36%) among Japanese children younger than five years of age (18). The difference in KD incidence between this report and the previous study can be attributed to race variations. The reported rates of KD are 10–20 cases per 100,000 for children under five years old among Caucasians, whereas it is 100–300 cases among children in East Asia (19).

In the present patients, we performed whole-exome sequencing to identify common mutations associated with PFAPA syndrome and KD. A heterozygous frameshift mutation, the 2-bp TT insertion at position rs140826611 in the *CARD8* gene, was identified in all patients with PFAPA syndrome complicated by KD. A previous study demonstrated that the identical *CARD8* gene variant (CARD8-FS) was significantly associated with the classical PFAPA syndrome (13). This study also showed that the frequency of CARD8-FS among patients with PFAPA syndrome (13.9%) was higher than that of healthy controls (3.2%). The minor allele frequency of CARD8-FS in the European population in the 1,000 Genomes Project was 5% (13). In previous European studies, the annual incidence of PFAPA syndrome was reported to be 2.3–2.6 cases per 10,000 children under five years old (20, 21). However, to the best of our knowledge, the incidence of PFAPA syndrome in children in East Asia, including Japan, has not been reported. To estimate the incidence of PFAPA syndrome in Japan, we inferred it from the ratio of PFAPA syndrome to KD in our hospital from January 1, 2016, to December 31, 2019. We chose a pre-pandemic period because the coronavirus disease pandemic indirectly influenced the diagnosis of both PFAPA syndrome and KD (22, 23). During this period, we diagnosed 36 patients with PFAPA syndrome and 235 patients with KD in our hospital. The average annual incidence ratio of the PFAPA syndrome to KD was 1:6.5 (our unpublished observation). Since the annual incidence of KD is 200–300 per 100,000 children under five years of age in Japan (18), that of PFAPA syndrome would be 3.1–4.6 per 10,000 children. These results indicate that the frequency of PFAPA syndrome might be higher in Japan than in Europe (20, 21).

CARD8 is a component of the NLRP3 inflammasome which negatively regulates inflammasome activation and the capacity to process IL-1β (24). The CARD8-FS variant lacks the ability to interact with NLRP3 and cannot regulate inflammasome assembly, and further cannot suppress caspase-1 cleavage (13). Excess inflammasome activation in patients with PFAPA syndrome carrying the CARD8-FS variant could be explained by the loss of the negative regulator function of CARD8 (13). In this context, it is interesting that a single-nucleotide polymorphism rs7248320 in the long non-coding RNA AC008392.1, located upstream of *CARD8*, is reported to be associated with KD (25). Furthermore, patients with KD carrying a polymorphism of *ITPKC* (rs28493229) showed increased production of active IL-1β and IL-18 related to activation of the NLRP3 inflammasome (10).

As activation of the NLRP3 inflammasome also occurs in patients with KD, we hypothesized that the CARD8-FS variant, which activates the NLRP3 inflammasome, may be a susceptibility gene for KD. Although the CARD8-FS variant has not been recognized as a susceptibility gene associated with KD, it could be an essential factor in the pathogenesis of both the PFAPA syndrome and KD. Therefore, we assessed 752 patients with KD, expecting that the frequency of CARD8-FS would be higher. However, the allele frequency of CARD8-FS was 13.0%, similar to that of the general Japanese population (12.9%) (26). As such, further studies are required to elucidate the mechanism underlying shared inflammatory dysregulation.

The present study had several limitations which should be mentioned. First, we enrolled only a small group of patients with PFAPA syndrome and KD in a limited region. Second, this was a single-center study, and the extrapolation of the frequency of patients with PFAPA syndrome deduced from the number of patients with KD therefore requires cautious interpretation.

## Conclusions

In the present study, we reported three cases of a rare combination of the PFAPA syndrome and KD carrying the CARD8-FS variant. Due to a higher prevalence of KD among patients with PFAPA syndrome and inflammasome activation in both PFAPA syndrome and KD, we hypothesized that the CARD8-FS variant might play an essential role in the pathogenesis of PFAPA as well as KD. However, we could not identify a difference in the variant frequency between patients with KD and the general Japanese population. The cause of KD is multifactorial and the frequency of KD among the Japanese population is much higher than that of PFAPA syndrome. As such, only a tiny proportion of patients with KD may have CARD8-FS as a causative factor, whereas others may have different factors.

## Data Availability

The datasets presented in this study can be found in online repositories. The names of the repository/repositories and accession number(s) can be found in the article/Supplementary Material.
